# In vitro Interleukin-7 treatment partially rescues MAIT cell dysfunction caused by SARS-CoV-2 infection

**DOI:** 10.1038/s41598-021-93536-7

**Published:** 2021-07-08

**Authors:** Satanay Hubrack, Maryam Ali Al-Nesf, Nourhen Agrebi, Christophe Raynaud, Mohammed Abu Khattab, Merlin Thomas, Tayseer Ibrahim, Salma Taha, Said Dermime, Maysaloun Merhi, Michal Kulinski, Martin Steinhoff, Patrick Tang, Bernice Lo

**Affiliations:** 1 Department of Human Genetics, Research Branch, Sidra Medicine, 26999 Doha, Qatar; 2 Allergy and Clinical Immunology Section, Hamad Medical Corporation, 3050 Doha, Qatar; 3 Center of Metabolism and Inflammation, Division of Medicine, Royal Free Campus, University College London, Rowland Hill Road, London, NW3 2PF UK; 4 Division of Infectious Disease, Hamad Medical Corporation, 3050 Doha, Qatar; 5 Pulmonary Division, Hamad Medical Corporation, 3050 Doha, Qatar; 6 Translational Cancer Research Facility, National Center for Cancer Care and Research, Translational Research Institute, Hamad Medical Corporation, 3050 Doha, Qatar; 7 Translational Research Institute, Hamad Medical Corporation, 3050 Doha, Qatar; 8 Weill Cornell Medicine, 24144 Doha, Qatar; 9 Weill Cornell Medicine, New York, NY 10021 USA; 10 Department of Dermatology, Hamad Medical Corporation, 3050 Doha, Qatar; 11 Department of Pathology, Sidra Medicine, 26999 Doha, Qatar; 12 College of Health and Life Sciences, Hamad Bin Khalifa University, 34110 Doha, Qatar

**Keywords:** Innate immune cells, Viral infection

## Abstract

MAIT cells have been shown to be activated upon several viral infections in a TCR-independent manner by responding to inflammatory cytokines secreted by antigen-presenting cells. Recently, a few studies have shown a similar activation of MAIT cells in response to severe acute respiratory coronavirus 2 (SARS-CoV-2) infection. In this study, we investigate the effect of SARS-CoV-2 infection on the frequency and phenotype of MAIT cells by flow cytometry, and we test in vitro stimulation conditions on the capacity to enhance or rescue the antiviral function of MAIT cells from patients with coronavirus disease 2019 (COVID-19). Our study, in agreement with recently published studies, confirmed the decline in MAIT cell frequency of hospitalized donors in comparison to healthy donors. MAIT cells of COVID-19 patients also had lower expression levels of TNF-alpha, perforin and granzyme B upon stimulation with IL-12 + IL-18. 24 h’ incubation with IL-7 successfully restored perforin expression levels in COVID-19 patients. Combined, our findings support the growing evidence that SARS-CoV-2 is dysregulating MAIT cells and that IL-7 treatment might improve their function, rendering them more effective in protecting the body against the virus.

## Introduction

Mucosal-associated invariant T (MAIT) cells are a sub-population of innate T lymphocytes with effector-like properties^1^. They are mainly found in the blood, lung, liver, and mucosa serving as sentinels against microbial and fungal infection^1,2^. Upon activation, they secrete pro-inflammatory cytokines and can kill bacteria or viral-infected cells by secretion of the cytolytic molecules granzyme B and perforin^3^. MAIT cells have been shown to be activated during human viral infections such as dengue virus, hepatitis C virus, and influenza virus^4^. MAIT cell activation correlates with disease severity in acute dengue infection^5^, and the reconstitution of MAIT cell levels in peripheral blood was suggested to have a positive outcome in HIV patients^6^. MAIT cells can be activated in viral infections in response to IL-12 or IL-15 together with IL-18^7,8^, and IL-7 is known to enhance the production of cytolytic molecules by these cells^8^. One study showed that the use of IL-7 alongside anti-retroviral therapy increased the number and frequency of MAIT cells in the peripheral blood of patients chronically infected with HIV^9^.

The effect of severe acute respiratory syndrome coronavirus 2 (SARS-CoV-2) infection on the immune system has been investigated in several studies; the most significant findings included a correlation between the severity of the disease, lymphopenia and elevated levels of certain cytokines^10,11^. It has been suggested that T cell exhaustion might be one of the factors leading to the low cell counts observed in critically ill patients^12^.

Recent studies investigated the activation of MAIT cells in SARS-CoV-2 infection, they found high activation and depletion of MAIT cells in COVID-19 patients^13–15^. One report associated the activation status of MAIT cells with disease severity and poor patient outcome^13^, while another recent study described altered function of MAIT cells in COVID-19 patients, due to an imbalance in IFN-α and IL-18^16^.

In this study, we focus on the effect of SARS-CoV-2 infection on the frequency, activity and phenotype of MAIT cells and investigate the capacity of IL-7 to enhance the function of MAIT cells in patients with COVID-19 in vitro.

## Materials and methods

### Statement

The study received approval from the local institutional review board of Hamad Medical Corporation (MRC-05-003). All study participants provided a written informed consent in accordance with the protocols of the study. All methods and protocols were performed in accordance with relevant guidelines and regulations.

### Subjects

Patients positive for SARS-CoV-2 by real-time RT-PCR who required ICU admission due to COVID-19 disease or disease complications were considered severely affected, while patients who tested positive and did not require ICU care were considered mildly affected. The control group was age, sex, and ethnicity matched healthy donors with no history of previous SARS-CoV-2 infection (Table S1). All SARS-CoV-2 positive patients recruited in this study were recruited and assigned as severe or mild patients within approximately one week (Mean ± SD = 5.1 ± 2.4 days) from positive real-time-PCR. None of the patients in the mild group developed severe symptoms later on. The severity of infection symptoms and clinical parameters for the patients included in this analysis is indicated in supplementary table 1 (Table S1). PBMCs were stored at − 80 °C and were not stored in liquid nitrogen post-ficoll.

### Peripheral blood mononuclear cells (PBMCs) isolation

Blood samples were collected in EDTA tubes within 7 days of admission. Peripheral blood mononuclear cells (PBMCs) were isolated from peripheral blood by Ficoll-Paque density gradient centrifugation (GE life sciences) within 24 h of collection, then stored at − 80 °C until further processing.

### Flow cytometry

Two flow cytometry panels were designed to quantify MAIT cell frequency and measure cell function. Frozen PBMC samples were thawed, washed, then stained with Live/Dead Fixable Aqua—400 (Molecular Probes). Fc receptors were then blocked using Human Fc Block (BD Biosciences). Cell surface staining was performed using directly conjugated antibodies in staining buffer (PBS containing 1% heat-inactivated FBS) for 30 min at 4 °C. Cells were then fixed in 4% paraformaldehyde solution for 20 min. Intracellular staining was performed after fixation and 15 min incubation in fix/perm buffer (BD Biosciences). Cells were incubated with the antibody mix in Perm/Wash buffer for 30 min at 4 °C, then washed in Perm/Wash buffer and re-suspended in staining buffer for data acquisition. Samples were acquired within 6 h of staining. Analyses were performed using FlowJo V10 and gates were based on fluorescence minus one (FMO) and presence of a clear positive population as shown in Figure S1.

### MAIT cell stimulations

PBMCs were cultured in a 96 well plate in advanced RPMI media (Gibco) containing 10% FBS. Cells were stimulated for 24 h with a mix of 10 ng/mL IL-12 (InvivoGen) and 100 ng/mL IL-18 (Biovision), or with 10 ng/mL IL-7 (Novus Biological) as previously described^17^. GolgiStop (BD Biosciences) was added to the samples for the last 6 h of stimulation.

### Antibodies

Anti-human CD3 PerCP (Clone: UCHT1), anti-human CD4 BV650 (Clone: RPA-T4), anti-human TCR V7.2 AF647 (Clone: 3C10), anti-human CD161 PE/Dazzle 594 (Clone: HP-3G10) and BV785 (Clone: HP-3G10), anti-human CD69 BV605 (Clone: FN50), anti-human CD8 APC/Fire 750 (Clone: SK1), anti-human TNF-α PE/Cyanine7 (clone: MAb1), anti-human IFN-γ AF488 (Clone: 4S.B3), anti-human perforin BV421 (Clone: DG9) and anti-human/mouse granzyme B PE (Clone: QA16A02) were from BioLegend Inc. Anti-human CD3 BV786 (Clone: SK7) was from BD Biosciences.

### Statistical analysis

Statistical analyses were performed using Prism Version 6 software (GraphPad), and p values < 0.05 were considered significant. The Mann–Whitney test was conducted to compare different groups. Multiple t-tests with Holm-Sidak’s correction were conducted for multiple comparisons. Comparisons between treatment conditions of samples from the same patient were done using two tailed paired t-test.

## Results

### SARS-CoV-2 infection causes MAIT cell activation and depletion in patients with severe symptoms

**Figure 1:**
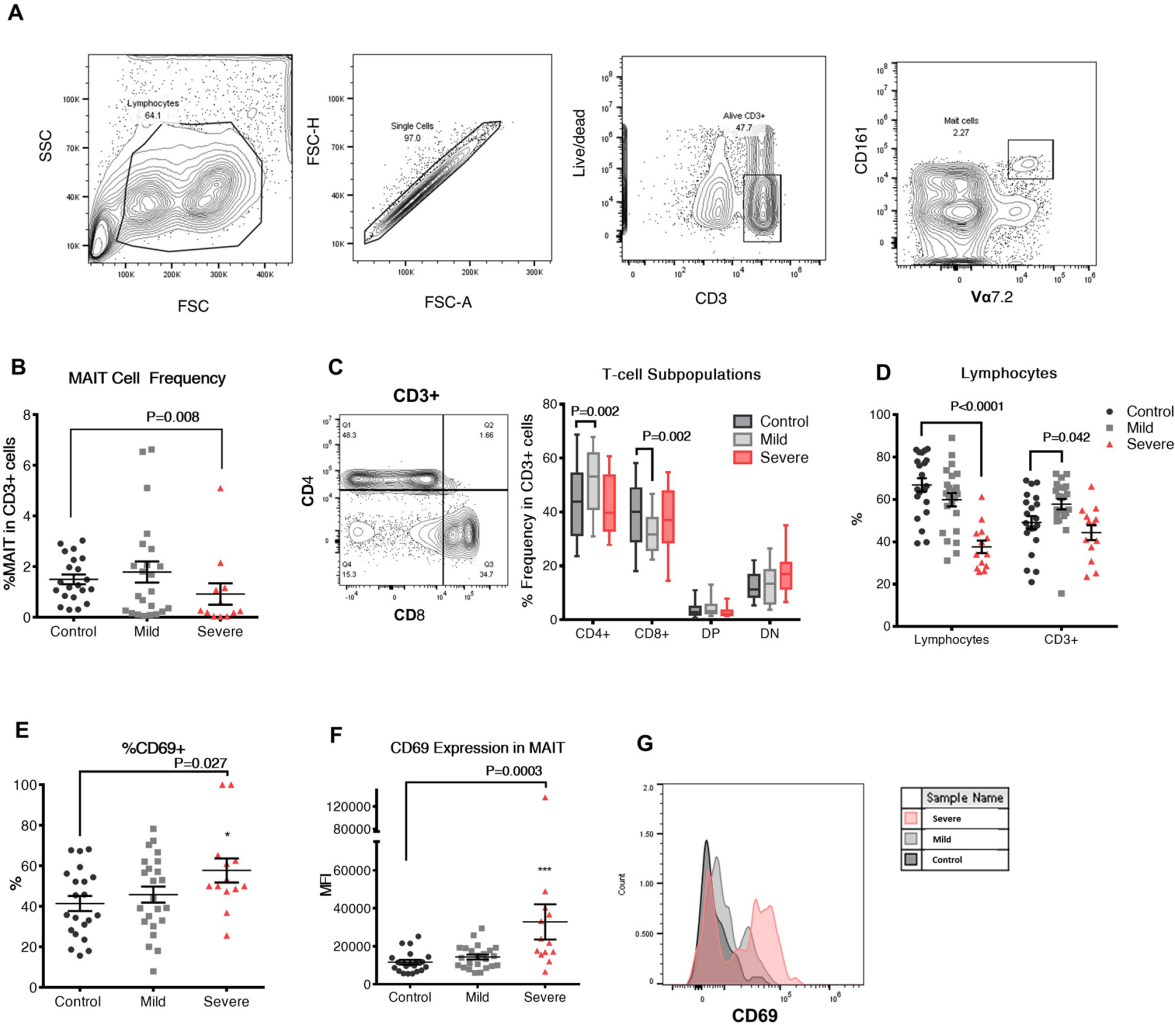
Prevalence of MAIT cells and other CD3+ populations in COVID-19 patients compared to uninfected individuals. (**A**) Gating strategy for identifying CD161 + Vα7.2 + MAIT cells in PBMCs done on a healthy control. (**B**) MAIT cell frequency in CD3+ population of PBMCs isolated from the mild and severe groups as compared to the control group. (**C**) Percentage of CD4 and CD8 double positive (DP) and double negative (DN) T-cell subpopulations in CD3+ cells of COVID-19 patients compared to unaffected individuals. (**D**) Percentage of the lymphocyte population identified in PBMCs isolated from peripheral blood of uninfected control individuals compared to COVID-19 mildly affected and severely affected patients and the prevalence of CD3+ cells in the lymphocyte population. (**E**) Percentage of MAIT cells expressing CD69 and (**F)** Mean fluorescence intensity (MFI) of CD69 in MAIT cells of healthy controls and COVID-19 patients reflecting the activation state of these cells. The Mann–Whitney test was conducted to compare each patient group with the control group in (**B**,**D**,**F**), error bars indicate the standard error of the mean. For (**C**,**E**), multiple t-tests with Holm-Sidak’s correction for multiple comparisons were conducted. (**G**) Histogram representation of CD69 expression in one healthy control, one mildly affected and one severely affected COVID-19 patient.

MAIT cells were identified as CD161 + Vα7.2 + T cells (Fig. 1A), and their frequencies were quantified in PBMCs isolated from 21 healthy individuals and 36 SARS-CoV-2 positive patients (23 mildly affected and 13 severely affected). Since MAIT cell frequencies were shown to decrease in adulthood^18,19^, samples in the control group were age and sex-matched. Levels of MAIT cells were significantly lower in patients in the severe group when compared to controls (0.92% ± 0.42%, 1.5% ± 0.19%; p = 0.008; Fig. 1B). While no significant difference was observed between the mild patient group and the controls, the frequency of MAIT cells in the mild group varied greatly from low MAIT frequency (< 1%; 0.31% ± 0.08%; n = 11) to normal or slightly elevated MAIT cell frequency (> 1%; 3.15% ± 0.55%; n = 12). We observed an increase in CD4+ cells and a decrease in CD8+ cells between patients in the mild group and controls, however, this difference was not seen between the control and severe patient groups (Fig. 1C). Therefore, no correlation between COVID infection and relative proportion of CD4+/CD8+ MAIT cells could be made. We did, however, observe severe lymphopenia in the severely affected patients (Fig. 1D). When comparing frequencies of CD4+ and CD8+ expressing MAIT cells between the control and the mildly affected group, we found a significant decrease in CD8+ MAIT cells while no change was observed in CD4+, double-positive and double-negative populations (Figure S2A).

To determine whether MAIT cells were activated in response to SARS-CoV-2 infection, we examined CD69 expression on MAIT cells isolated from patients and controls. Our data show a significant upregulation of MAIT cell activation in the severe patient group compared to the healthy control group (Fig. 1E,F).

### MAIT cells are functionally affected in COVID-19 patients, and their functionality can be partially rescued by IL-7 treatment

**Figure 2:**
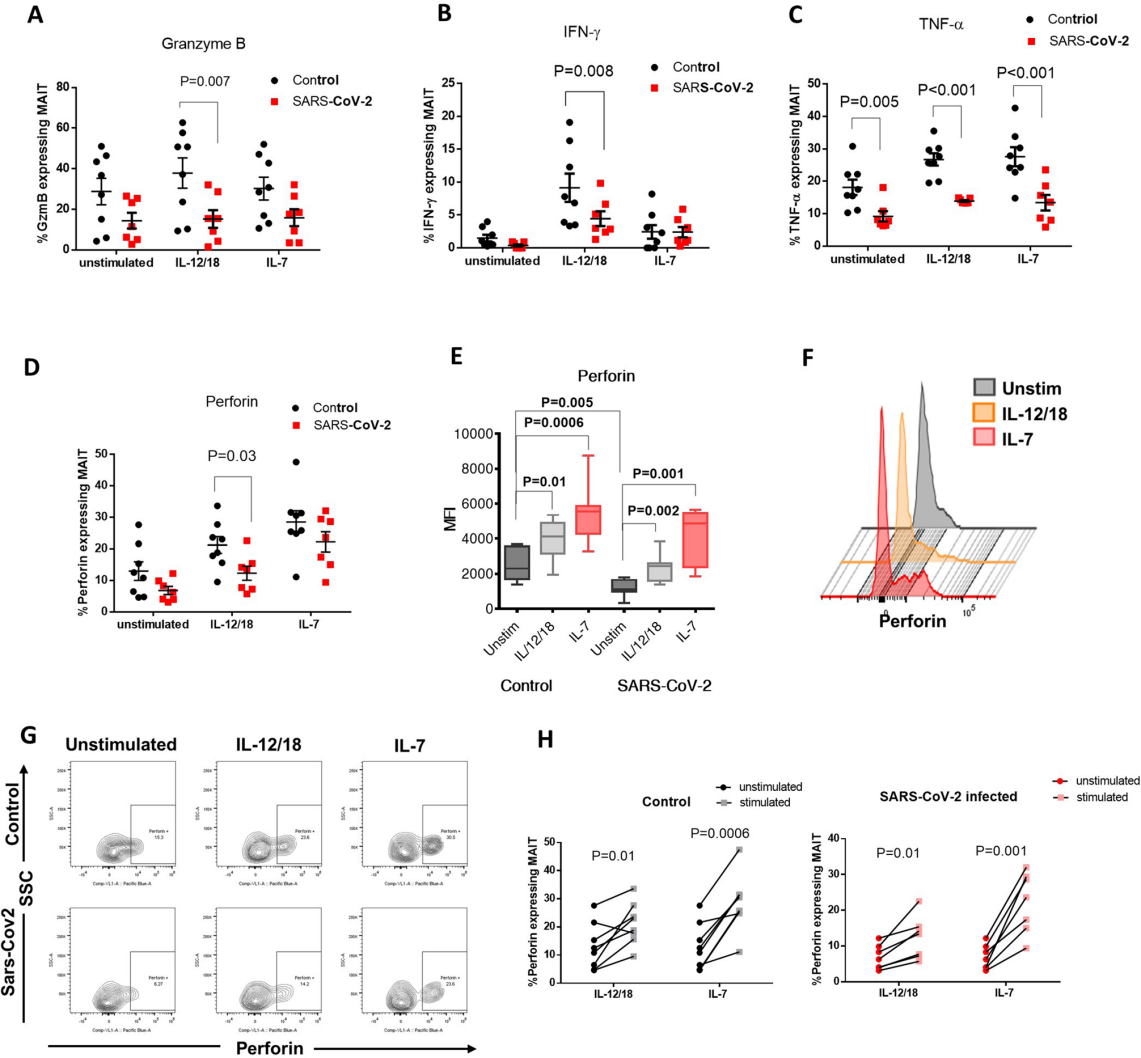
Effect of SARS-CoV-2 infection on the function of MAIT cells measured by the expression levels of cytokine and cytolytic proteins upon in vitro stimulation. Frequency of MAIT cells expressing (**A**) granzyme B (**B**) IFN-γ, (**C**) TNF-α and (**D**) perforin in PBMCs isolated from the peripheral blood of COVID-19 patients (presenting with mild symptoms) compared to uninfected individuals with or without stimulation with a combination of IL-12 and IL-18 or IL-7 alone. Multiple t-tests with Holm-Sidak’s correction for multiple comparisons were conducted to compare the SARS-CoV2 infected group with the control uninfected group; bars show the standard error of the mean. (**E**) Mean fluorescence intensity (MFI) of perforin in MAIT cells isolated from control and infected individuals. Mann–Whitney t-test was conducted to compare the unstimulated patients and control groups, and paired t-tests were used to compare stimulated and unstimulated cells within each group, bars show maximum and minimum values (**F**) Histogram representation of perforin expression in MAIT cells of a mildly affected individual that were either unstimulated or incubated with IL-12/18 nor IL-7 for 24 h. (**G)** Flow cytometric analysis of MAIT cells expressing perforin, showing an example of a control individual and a SARS-CoV2 infected patient. (**H**) Percentage of MAIT cells expressing perforin in the control and patient groups with or without stimulation with IL-12/18 or IL-7 alone. Paired t-tests were conducted to determine statistical significance between stimulated and unstimulated groups.

To further investigate the effect of SARS-CoV-2 infection on MAIT cell function, we quantified the frequencies of MAIT cells expressing cytokines (IFN-γ and TNF-α) and cytolytic molecules (granzyme B and perforin) in 8 uninfected individuals and 7 COVID-19 patients. Due to the significant lymphopenia of severely affected COVID-19 patients (Fig. 1D), these subsequent analyses could only be conducted on the cells of mildly affected individuals. Since MAIT cells were shown to be activated in response to a combination of IL-12 and IL-18 upon viral infection^7^, we cultured PBMCs from uninfected controls and COVID-19 patients in media containing IL-12 and IL-18 for 24 h to mimic MAIT cell stimulation during viral infections. This stimulation was effective in the control group (Figure S2B). Without stimulation, our data show a substantial decrease in the percentage of MAIT cells expressing granzyme B and perforin and a significant decrease in TNF-α (p = 0.005) between the control and SARS-CoV-2+ groups Fig. 2A,C,D).

Upon stimulation with IL-12/IL-18, the percentage of Granzyme B (p = 0.007), IFN-γ (p = 0.008), TNF-α (p < 0.001) and Perforin (p = 0.03) expressing MAIT cells was significantly lower in COVID-19 patients compared to control uninfected individuals (Fig. 2A–D). We also observed a significantly lower staining intensity (p = 0.005) for perforin in the MAIT cells of the COVID-19 group compared to the control group (Fig. 2E).

To attempt to rescue the cytotoxic function of MAIT cells in COVID-19 patients, we treated PBMCs with IL-7 for 24 h. IL-7 stimulation of the control group only increased the percentage of perforin and TNF-α expressing MAIT cells (Fig. 2G,H and Figure S3). IL-7 treatment did not significantly increase the frequency of MAIT cells expressing TNF-α or granzyme B in COVID-19 patients (Figure S3A,D,C,F), but it successfully increased both the perforin expression level (MFI) (p = 0.001, Fig. 2E,F) and the frequency of MAIT cells expressing perforin (p = 0.001, Fig. 2G,H). IL-7 treatment raised the frequency of perforin expressing MAIT cells from COVID-19 patients to a level comparable to the IL-12/18 stimulated control groups (Fig. 2D).

IL-7 also significantly increased the frequency of IFN-γ expressing cells in COVID-19 patients but to a lower level than that achieved by IL-12/IL-18 stimulation (Figure S1B,E).

## Discussion

Our results show a significant decrease in MAIT cell frequency in severely affected COVID-19 patients (Fig. 1B). Similar depletion of MAIT cells has been reported for other viral infections such as measles and HIV^20,21^. The depletion of MAIT cells upon measles infection was attributed to measles-induced apoptosis^20^, while the decline of MAIT cell levels in HIV-infected patients was shown to be due to the activation and exhaustion of these cells^22^. In this study, we found the levels of CD69 expression to be significantly higher in severely affected COVID-19 patients (Fig. 1E,F). This increase indicates a higher activation status of these cells. These findings are consistent with recent reports showing MAIT cell loss along with heightened activation in COVID-19 patients^14,15,23^, suggesting that the observed MAIT cell depletion might be due to the elevated activation and subsequent exhaustion of these cells. Since MAIT cells act as the first line of defense against various pathogens, their depletion might lead to weakened mucosal immunity, leaving patients more vulnerable to secondary infections caused by opportunistic pathogens^24^.

Upon activation, MAIT cells gain cytotoxic abilities and release granzyme B and perforin to kill target cells^25^. To investigate the effect of SARS-CoV-2 on the cytotoxic ability of MAIT cells, we carried out in vitro stimulation experiments using IL-12/IL-18 treatment as a positive control for activation since it has been previously demonstrated to induce MAIT cells in vitro^26^. Our results show that SARS-CoV-2 infection significantly lowers the frequency of MAIT cells expressing Granzyme B and TNF (Fig. 2A,C), and also the frequency of perforin expressing cells upon IL-12/18 activation (Fig. 2D). These findings indicate that, in addition to MAIT cell depletion, SARS-CoV-2 infection disrupts MAIT cell function, diminishing their ability to protect the body against pathogens. On the other hand, restoring the function of these cells can be of great benefit in boosting the defense against potential secondary infections. We found that 24-h stimulation with IL-7 could successfully restore the frequency of perforin expressing MAIT cells of COVID-19 patients, which suggests that IL-7 treatment might be beneficial in arming MAIT cells affected by SARS-CoV-2 infection.

During the course of publication of this manuscript, more studies were published, either confirming the observed effect of COVID-19 on MAIT cells we describe in this study^23^, or linking MAIT cell cytotoxicity to poor COVID-19 patient outcome^13,16^. While our findings show a substantial decrease in granzyme B production in MAIT cells of COVID-19 patients, these studies show an increase in granzyme B expression by MAIT cells in SARS-COV2 infected patients. One explanation might be that MAIT cells analyzed in these publications are activated in a TCR-dependent manner by secondary bacterial infections, while in our cohort none of the patients included in the functional studies presented with bacterial co-infection. The presence of a secondary infection also leads to increased disease severity. This calls for more studies investigating the effect of SARS-CoV2 infection on MR1-dependent MAIT cell activation in response to secondary bacterial infections.

In vitro IL-7 treatment was previously shown to restore effector functions in MAIT cells isolated from HIV-1 infected patients^17^, and subcutaneous injection of IL-7 could restore the levels of MAIT cells in these patients^9^. IL-7 immunotherapy is currently being evaluated as a treatment to reverse the lymphopenia in COVID-19 patients and was shown to restore lymphocyte count in critically ill COVID-19 patients without worsening pulmonary injury and inflammation^27^. Another case study showed a significant improvement in lymphocyte count after IL-7 treatment in a 74 years old COVID-19 patient^28^. Our findings suggest that IL-7 therapy may also improve the function of MAIT cells affected by SARS-CoV-2 infection. In this work, we analyzed the in vitro effect of IL-7 on MAIT cells of only mildly affected patients as we had limited samples from the severely affected patients which presented with severe lymphopenia and thus had very few MAIT cells still present. Nonetheless, as discussed by Monneret et al., treatment of patients at an earlier stage before the development of further complications might be more beneficial for the patient. Therefore, testing the possibility of recovering the MAIT cells function during mild disease may demonstrate the benefits of treating at an early stage of disease. One drawback of immunostimulatory therapies for COVID-19 is the increased risk of inducing an exaggerated immune response triggering a cytokine storm^29^. Since we showed that IL-7 treatment augmented perforin levels without significantly increasing TNF-α and IFN-γ production, this may indicate that IL-7 therapy might enhance the functionality of MAIT cells without contributing to the cytokine storm associated with severe COVID-19. We also postulate that IL-7 could be used as a precautionary treatment in patients considered high risk for developing severe symptoms with SARS-CoV-2 infection.

IL-7 signaling plays important physiological roles and is implicated in inflammatory diseases and cancer^30^, which emphasizes the need for more clinical trials to evaluate the benefits and the possible drawbacks of using it as a treatment for critically ill COVID-19 patients.

## Supplementary Information

Supplementary Information.

## Data Availability

Not applicable.
