# Supplementary material for: In vitro Interleukin-7 treatment partially rescues MAIT cell dysfunction caused by SARS-CoV-2 infection
Source: Sci Rep. 2021 Jul 8;11:14090. doi: 10.1038/s41598-021-93536-7 (PMC8266862; doi:10.1038/s41598-021-93536-7)
Supplement: Supplementary file 1 — Supplementary Information. [file 41598_2021_93536_MOESM1_ESM.docx]

**Supplementary Material.**

**Supplementary Material.**

**Table S1. Demographics and clinical characteristics of control and patients with COVID-19.**

|  | **Healthy Controls**  **(N=21)** | **COVID-19 (mild)**  **(N=23)** | **COVID-19 (severe)**  **(N=13)** |
| --- | --- | --- | --- |
| **Sex** |  |  |  |
| **Male: Female** | 20:1 | 21:2 | 12:1 |
| **Age** |  |  |  |
| **Mean±SD** | 38±7.32 | 38.39±10.47 | 41.15±12.4 |
| **Range** | 23-50 | 18-55 | 24-62 |
| **Symptoms n (%)** |  |  |  |
| **Asymptomatic** |  | 5 (21.7) | 0 (0) |
| **Fever** |  | 13 (56.5) | 12 (92.3) |
| **Coryza (flu)** |  | 13 (56.5) | 7 (53.9) |
| **Anosmia & dysgeusia** |  | 1 (4.3) |  |
| **Cough** |  | 16 (69.6) | 12 (92.3) |
| **Difficulty of breath** |  | 4 (17.4) | 12 (92.3) |
| **Gastrointestinal symptoms** |  | 4 (17.4) | 2 (15.4) |
| **Organs affected n (%)** |  |  |  |
| **Lung (Pneumonia)** |  | 21 (91.3) | 13 (100) |
| **Heart (ACS- NSTEMI*)** |  | 0 (0) | 1 (7.7) |
| **Liver (elevated liver enzymes)** |  | 3 (13.0) | 2 (15.4) |
| **Kidneys (acute kidney injury)** |  | 0 (0) | 1 (7.7) |
| **Multiorgan failure** |  | 0 (0) | 1 (7.7) |
| **Outcome n (%)** |  |  |  |
| **Alife and back to normal** |  | 23 (100) | 12 (92.3) |
| **Died** |  | 0 (0) | 1 (7.7) |
| **Bacterial co-infections** |  | 0 (0) | 0 (0) |
| **Interval from 1st positive PCR test**  **to blood collection (Mean±SD days)** |  | 4.78±2.21 | 5.54±2.76 |

***ACS-NSTEMI: Acute Coronary Syndrome without ST-segment elevation.**

**Some patients may have more than one symptom.**

**Some patients were asymptomatic; however, found accidentally to have pneumonia by chest x-ray in the mild group and recovered with no treatment.**

**Supplementary Figures**

**
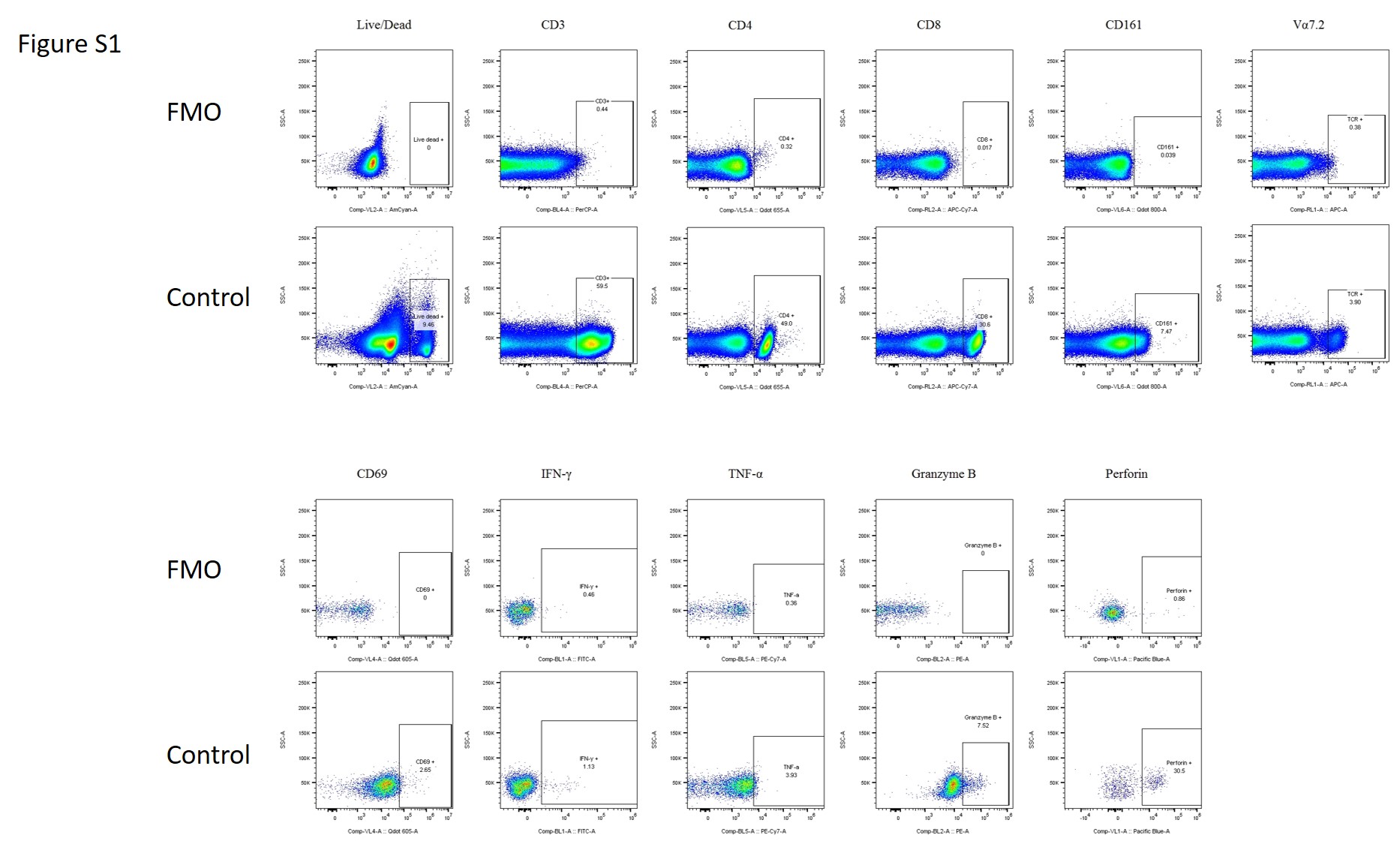
Figure S1.** FMO (top lane) and corresponding healthy control donor (bottom lane) staining for each marker used. Positive/negative gating was defined based on FMO and separation of clear positive and negative populations in the control sample as shown.


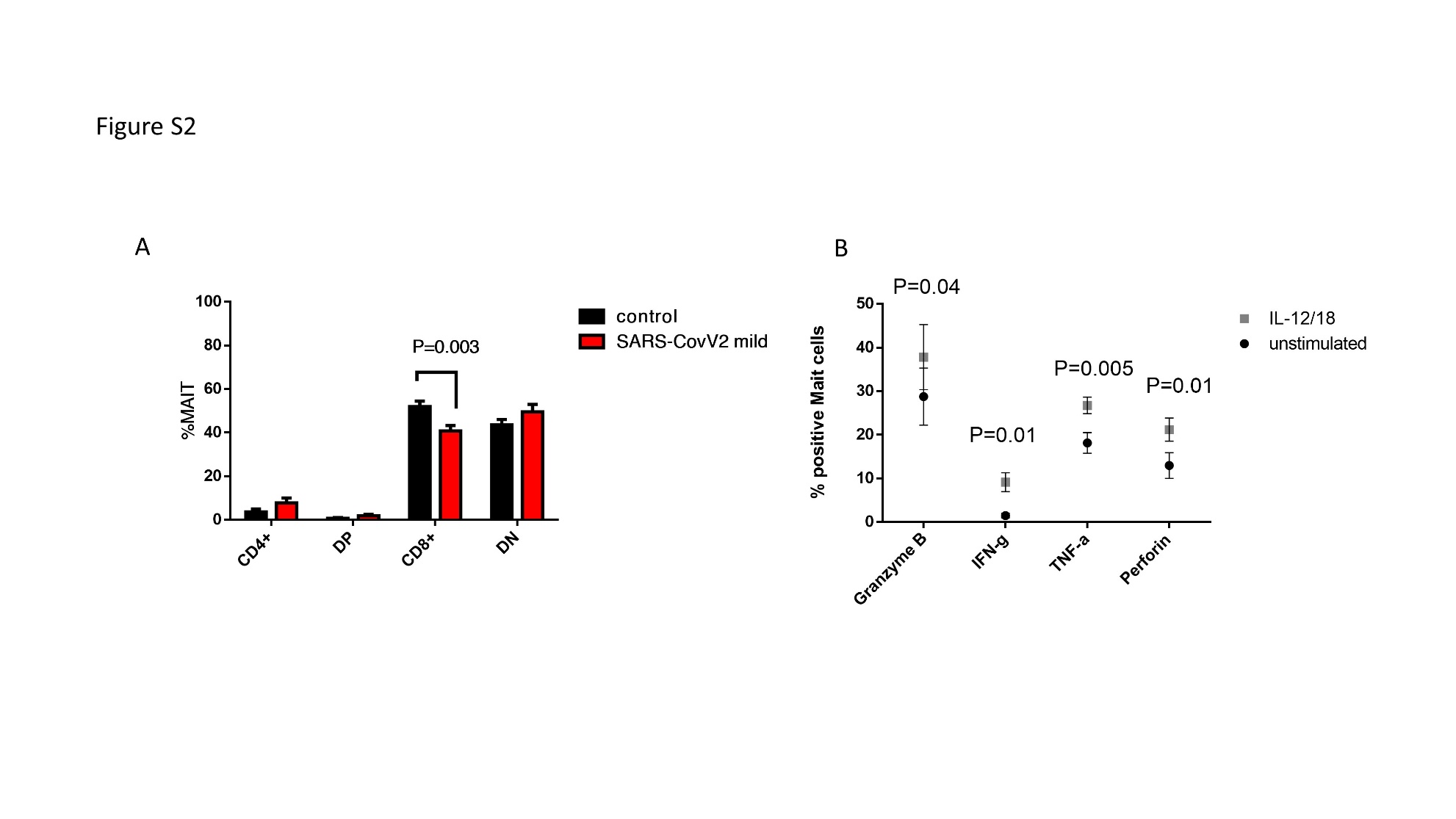


**Figure S2. (A)** Frequency of CD4+, CD8+, double positive and double negative MAIT cell populations in control and mildly affected COVID-19 patients. Multiple t-tests with Holm-Sidak’s correction for multiple comparisons were conducted; bars show the standard error of the mean. **(B)** Percentage of MAIT cells expressing Granzyme B, TNF-α, IFN-γ or Perforin with or without stimulation by IL-12/18. Paired t-tests were conducted to determine statistical significance between stimulated and unstimulated groups.


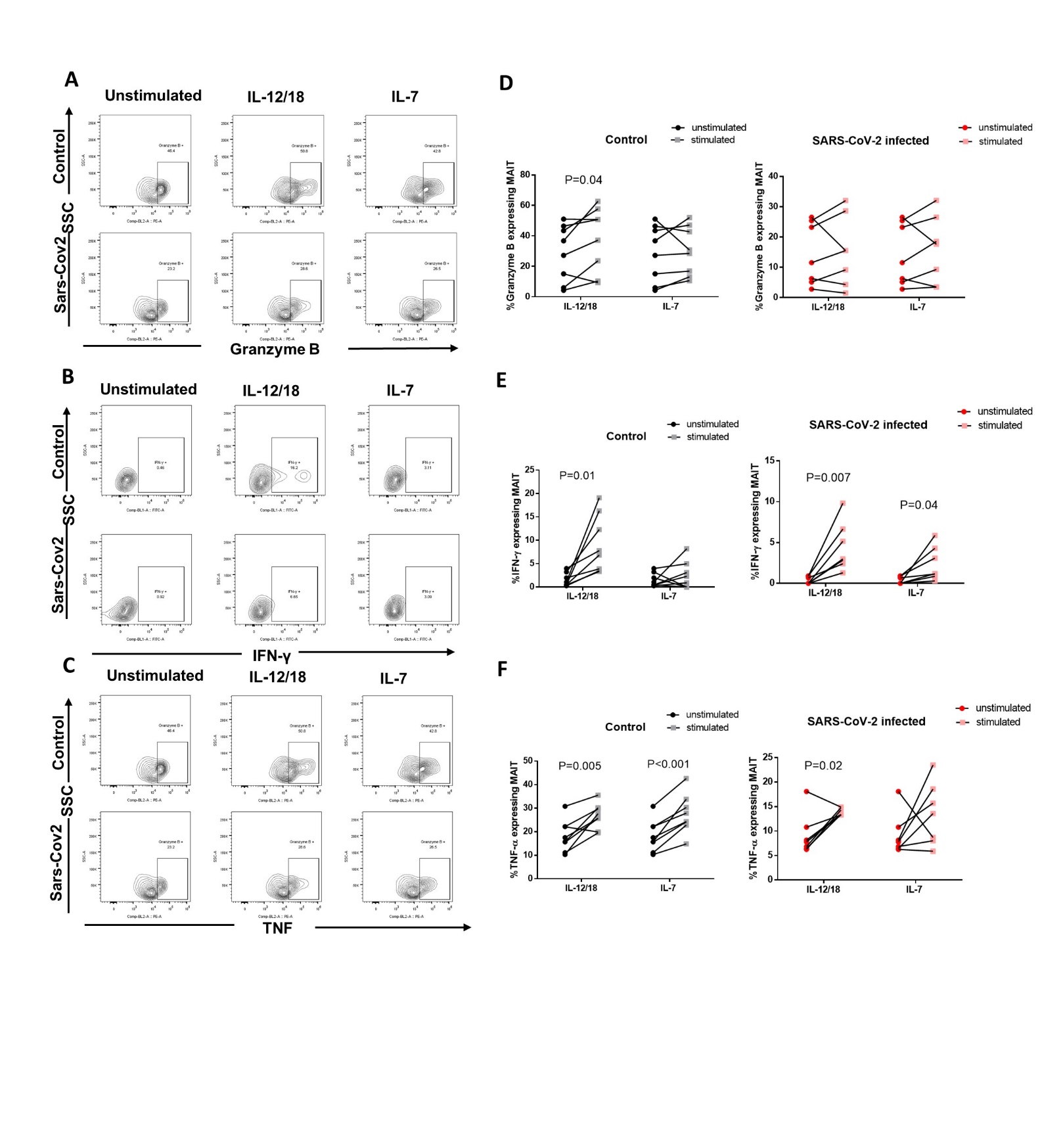


**Figure S3.** Flow cytometric analysis of MAIT cells expressing granzyme B, IFN-γ and TNF-α. **(A-C)** each showing an example of a control individual and a SARS-CoV2 infected patient. **(D-F)** Percentage of MAIT cells expressing granzyme B, IFN-γ and TNF-α in the control and patient groups with or without stimulation with IL-12/18 or IL-7 alone. Paired t-tests were conducted to determine statistical significance between stimulated and unstimulated groups.
